# Can laparoscopic common bile duct exploration be performed without any drainage? A propensity score–matched study

**DOI:** 10.20452/wiitm.2024.17909

**Published:** 2024-11-07

**Authors:** Chufa Zheng, Weifeng Wang, Qiquan Peng, Yunheng Peng, Xiaozhong Wang

**Affiliations:** Department of General Surgery, Shantou Central Hospital, Shantou, China; Department of Gastrointestinal Surgery, Sun Yat‑sen Memorial Hospital, Sun Yat‑sen University, China

**Keywords:** abdominal drainage, choledocholithiasis, laparoscopic common bile duct exploration, primary suture, propensity score matching

## Abstract

**INTRODUCTION:**

Although laparoscopic common bile duct exploration (LCBDE) is considered a safe and effective method for treating choledocholithiasis, the absence of any biliary or abdominal drainage during surgery remains controversial.

**AIM:**

This paper aims to investigate the feasibility and safety of LCBDE without drainage, particularly abdominal drainage.

**MATERIALS AND METHODS:**

This retrospective analysis included 499 patients who underwent LCBDE with primary closure of the common bile duct and without any kind of biliary drainage during surgery. In 322 individuals, the surgery involved routine abdominal drainage (drainage group), whereas in 177 cases, no abdominal drainage was performed (nondrainage group). Baseline characteristics of the 2 groups were compared, followed by propensity score matching (PSM) to balance confounding factors. We compared effect indicators and complication rates between both groups.

**RESULTS:**

After PSM, each group included 124 patients. There were no significant differences between the 2 groups in terms of overall and individual complication rates, except for a lower incidence of hyperamylasemia in the nondrainage group. The surgery time, duration of postoperative antibiotic use, and the total and postoperative length of hospital stay was significantly shorter in the nondrainage group. Similarly, the total hospitalization cost and postoperative usage of analgesics and antispasmodics were also considerably lower in the nondrainage group.

**CONCLUSIONS:**

Nondrainage LCBDE is associated with shorter recovery time and better patient outcomes, as compared with procedures involving abdominal drainage. In suitable cases, this approach is completely safe and feasible.

## INTRODUCTION 

Currently, there are 2 main surgical approaches to managing common bile duct (CBD) stones combined with gallbladder stones. The first approach is laparoscopic CBD exploration (LCBDE) combined with laparoscopic cholecystectomy (LC). The second approach is endoscopic retrograde cholangiopancreatography (ERCP) followed by LC. Classification of LCBDE comprises 3 major categories: simple primary closure, T‑tube drainage, and primary closure combined with internal drainage. The last one is performed with the use of a biliary stent or external drainage, such as preoperative or intraoperative endoscopic nasobiliary drainage (ENBD) or percutaneous transhepatic cholangial drainage (PTCD). LCBDE is an important and minimally invasive treatment method for patients with choledocholithiasis. Single‑stage LCBDE+LC is superior to 2‑stage ERCP followed by LC in terms of technical success, duration and cost of hospitalization, and complication rate (eg, pancreatitis, bleeding, duodenal perforation).^1^^;^^2^^;^^3^^;^^4^^;^^5^^;^^6^ LCBDE with T‑tube drainage remains a classic method for managing CBD stones, as it prevents bile leakage and bile duct stricture. However, its safety is questionable, as T‑tube placement may lead to complications, such as retrograde bacterial infection, electrolyte imbalance, and bile peritonitis if the T‑tube is displaced.^7^^;^^8^ Additionally, it can cause inconvenience for patients in their daily lives. Primary CBD closure has been proven safe^9^ and might be performed as a priority alternative to T‑tube drainage for choledocholithiasis.^8^^;^^10^ Some studies have reported that primary duct closure with bile duct stent insertion^11^ or intraoperative ENBD^12^ shows better results in terms of postoperative hospital stay and hospitalization costs. However, not only may biliary stents, T‑tube drainage, or any kind of biliary drainage require additional procedures, but also neither of these approaches provides any added value for choledochotomy closure, and they may even be charged with specific morbidity.^13^ In the case of patients with primary closure during LCBDE, a peritoneal cavity drainage tube is routinely placed in the gallbladder fossa to reduce the risk of bile leakage. ^14^

## AIM 

There are limited data on whether patients can undergo LCBDE without any kind of drainage, including biliary drainage (such as T‑tube, biliary sent, ENBD, or PTCD) and peritoneal drainage. This retrospective study aimed to evaluate the safety and feasibility of LCBDE without any kind of drainage for individuals with choledocholithiasis.

## MATERIALS AND METHODS 

### General information 

We retrospectively analyzed patients with choledocholithiasis who underwent LCBDE with primary closure and without any kind of biliary drainage during surgery. The participants were treated at the General Surgery Department of our hospital between December 2020 and December 2023. The study population was further divided according to the use of abdominal drainage during LCBDE (drainage group vs nondrainage group). Preoperative general characteristics of the 2 groups, including sex, age, chronic diseases (hypertension, diabetes, pulmonary infection), preoperative acute cholangitis, and preoperative biliary pancreatitis were recorded. The collected laboratory data included preoperative levels of total bilirubin (TBIL), direct bilirubin (DBIL), alanine aminotransferase, aspartate aminotransferase, white blood cell count (WBC), neutrophil count, platelet count, Creactive protein, and procalcitonin. Imaging data included abdominal ultrasound, computed tomography (CT), or magnetic resonance cholangiopancreatography (MRCP). Based on the imaging findings, the CBD diameter along with the size and number of CBD stones were assessed and recorded by radiology specialists. The outcome measures of this study included surgical duration, hospitalization cost, total length of hospital stay, postoperative hospital stay, duration of postoperative antibiotic use, postoperative usage of antispasmodics and analgesics, and incidence of postoperative complications. The analyzed postoperative complications comprised bile leakage, stone recurrence, CBD stricture, postoperative pancreatitis, hyperamylasemia, peritoneal effusion, pleural effusion, pulmonary infection, and deep vein thrombosis (DVT) of the lower extremities.

### Inclusion criteria 

All patients were preoperatively diagnosed with CBD stones, with or without gallbladder stones, using abdominal ultrasound, CT, or MRCP. All extrahepatic bile duct stones were completely removed, with the distal CBD and duodenal papilla remaining unobstructed, and the sphincter of Oddi functioning well. Patients had complete clinical data, including hospitalization records, medical recommendations, examination results, and follow‑up information.

### Exclusion criteria 

The following exclusion criteria were applied: presence of intrahepatic bile duct stones, unremoved stones, or distal CBD strictures identified during intraoperative cholangioscopy, Mirizzi syndrome, Lemmel syndrome (periampullary duodenal diverticulum), history of multiple upper abdominal surgeries resulting in severe abdominal adhesions, acute suppurative cholecystitis, preoperative acute severe cholangitis or severe pancreatitis, concomitant malignant tumors (not limited to the liver, gallbladder, CBD, duodenum, or pancreas), and severe systemic or organic diseases, such as lupus nephritis, diabetic ketoacidosis, liver failure, and severe cardiovascular diseases.

### Definitions 

Postoperative complications were defined as any deviation from the normal post-operative course, including asymptomatic complications. Complications were analyzed according to the Clavien–Dindo classification.^15^ As per the International Study Group of Liver Surgery,^16^ bile leakage was defined as a bilirubin concentration in the drainage fluid at least 3 times the serum bilirubin concentration on or after post‑operative day 3, or the need for interventional drainage or surgical intervention due to bilious collections or bile peritonitis. The severity of bile leakage is classified into 3 groups: grade A requires no additional interventions, grade B requires active therapeutic interventions but can be managed without reoperation (eg, percutaneous drainage), and grade C requires reoperation. Acute cholangitis was defined in accordance with the 2018 Tokyo guidelines.^17^ We adopted the definition of acute pancreatitis as per the revised Atlanta classification from the 2012 International Consensus Guidelines.^18^ Postoperative hyperamylasemia was understood as postoperative serum amylase level greater than 115 U/l in patients with normal preoperative serum amylase values (15–115 U/l, as per the reference range adopted in our hospital). We defined recurrent CBD stones as stones detected in the CBD more than 6 months after complete removal of initial CBD stones.^19^ Postoperative usage of antispasmodics and analgesics was understood as routinely administered appropriate doses of antispasmodics and analgesics on the first postoperative day to accelerate recovery and encourage early mobilization. If the patient reported intolerable pain, the attending physician or on‑duty physician administered antispasmodics or analgesics within safe limits, as needed. The commonly used analgesics at our center include tramadol, flurbiprofen axetil, and parecoxib sodium. The commonly used antispasmodics include drotaverine and racanisodamine hydrochloride. Each medication administration followed the dosage recommended in the summary of product characteristics (with dosage adjustments for the elderly, children, or patients with liver or kidney insufficiency). Each administration was considered 1 treatment unit.

### Surgical procedures 

LCBDE with primary closure was performed by specialists with over 5 years of experience in hepatobiliary or laparoscopic surgery. Their job positions varied from attending physician to chief surgeon. During LCBDE, a standard 4‑port technique was used. After the patient was placed in the supine position, 2 10‑mm trocars were respectively inserted: the one above the umbilical region was used for the camera, whereas the other one placed the subxiphoid area served as the main working port and choledochoscope. The other 2 5‑mm trocars were inserted in the right anterior axillary line and the right midclavicular area, respectively, for the auxiliary working port. During the surgery, standard cholecystectomy was also performed in all but 43 patients (8.6%) who had undergone cholecystectomy in the past. Longitudinal choledochotomy was made in the anterior aspect of the CBD. Stones were removed by applying saline irrigation and a stone retrieval basket. A 5‑mm flexible choledochoscope (Olympus, Tokyo, Japan) was routinely used to examine the clearance of the distal and proximal CBD and the intrahepatic bile duct. No intraoperative cholangiography was performed. The choledochotomy incisions were primarily closed by interrupted suture using Vicryl 3–0 or 4–0 suture material (Ethicon Company, New Brunswick, New Jersey, United States). As in most hospitals, we routinely place a drainage tube at the Winslow foramen after LCBDE and remove it if there is no bile leak. In the cases without abdominal drainage tube placement, the following criteria must be met: confirmation of the complete removal of the stones in the bile duct by intraoperative cholangioscopy, accurate suturing of the CBD, and minimal abdominal contamination during surgery or adequate abdominal irrigation. If these requirements are fulfilled, we consider not placing an abdominal drainage tube after LCBDE.

### Follow-up 

All patients underwent long‑term follow‑up of at least 6 months. During the first 6 months postsurgery, follow‑up was primarily conducted in the outpatient department and included routine blood tests, liver function tests, ultrasound, CT, or MRCP. We reviewed all relevant data from the electronic medical record system, both inpatient and outpatient, and investigated the treatment process for any biliary diseases. After 6 months, we scheduled regular follow‑up phone calls to check whether the patients experienced any symptoms related to biliary diseases or had any consultations at hospitals due to biliary conditions.

### Ethics statement 

This is a retrospective study, with all data derived from previously existing clinical records. It does not involve any disclosure of patient personal data or intervention type. Therefore, it did not require approval from an ethics committee or written informed consent from the participants. This study strictly adheres to data anonymization and privacy protection regulations. We followed the ethical principles for clinical research set out in the Declaration of Helsinki.

### Statistical analysis 

Data analysis was performed using SPSS software, version 27.0.1 (IBM Corp., Armonk, New York, United States). To check for confounding factors and reduce selection bias, propensity score matching (PSM) was conducted. Propensity scores were estimated using a non‑parsimonious multivariable logistic regression model,^20^ with the baseline characteristics listed in used as covariates. Matching was performed using a 1:1 protocol without replacement (greedy matching algorithm), with a caliper width equal to 0.2 of the standard deviation of the logit of the propensity score.^21^ Standardized mean differences (SMDs) of all baseline covariates were calculated before and after matching to evaluate balance. An SMD of less than 10% for a given covariate indicated a relatively small imbalance. ^22^

Continuous variables which followed a normal distribution were presented as mean (SD), while those that did not were presented as median and interquartile range (IQR). Categorical variables were represented as counts or percentages. To compare the continuous variables that followed a normal distribution, the independent sample t test or corrected t test was used. For those not following a normal distribution, the Mann–Whitney test was applied. Categorical variables were compared using the χ^2^ test, the χ^2^ test with the continuity correction, or the Fisher exact test. A 2‑sided P value below 0.05 was considered significant.

## RESULTS 

### Comparison of baseline data 

**Figure 1:**
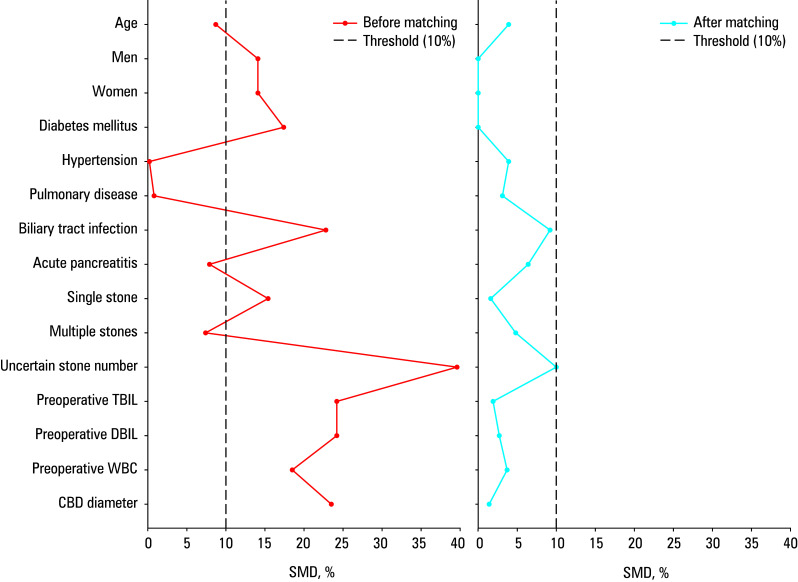
Baseline patient characteristics before and after propensity score matching

Abbreviations: see TABLE 1

Between December 2020 and December 2023, a total of 891 cholelithiasis surgeries were performed in our center (excluding simple cholecystectomies), of which were 863 laparoscopic procedures and 28 were open surgeries. Conversion to open surgery was required in 6 cases due to dense abdominal adhesions that prevented normal tissue dissection under laparoscopy. One patient died of cardiac arrest caused by severe aortic stenosis. Of the 891 surgeries, 93 involved T‑tube drainage, while simple primary closure of the CBD was performed in 798 individuals. A total of 499 patients met the inclusion criteria. All of them underwent LCBDE with primary closure of the CBD and without any kind of biliary drainage (such as T‑tube, biliary stent, ENBD, etc.) during surgery. Of the 499 patients, 177 underwent LCBDE without abdominal drainage, whereas routine abdominal drainage was performed in 322 participants. details the baseline characteristics of the groups before and after PSM, including sex, age, comorbidities (hypertension, diabetes, pulmonary infection, preoperative acute cholangitis, preoperative biliary pancreatitis), preoperative laboratory data (TBIL, DBIL, and WBC), and imaging data (CBD diameter, number of CBD stones). Prior to PSM, several baseline variables differed between the 2 groups, including sex (SMD, 14.1%), prevalence of diabetes (SMD, 17.4%) and preoperative acute cholangitis (SMD, 22.8%), CBD diameter (SMD, 23.5%), number of CBD stones (SMD, 15.4%), preoperative TBIL (SMD, 24.2%), preoperative DBIL (SMD, 24.2%), and preoperative WBC (SMD, 18.5%). After PSM, 124 patients in the nondrainage group were matched with 124 individuals in the drainage group. After matching, SMDs for all variables were less than 10%, indicating minimal differences between the 2 groups TABLE 1 ;Figure 1.

**TABLE 1:** Baseline patient characteristics before and after propensity score matching

Parameter		Before matching		SMD	After matching		SMD, %
		Drainage group (n =322)	Nondrainage group (n= 177)		Drainage group (n = 124)	Nondrainage group (n = 124)	
Age, y, mean (SD)		62.7 (13.49)	61.53 (14.51)	8.7	62.42 (13.45)	61.9 (13.86)	3.9
Sex	Men	170 (52.8)	81 (45.8)	14.1	63 (50.8)	63 (50.8)	0
	Women	152 (47.2)	96 (54.2)	14.1	61 (49.2)	61 (49.2)	0
Comorbidities	Diabetes mellitus	60 (18.6)	21 (11.9)	17.4	15 (12.1)	15 (12.1)	0
	Hypertension	73 (22.7)	40 (22.6)	0.2	28 (22.6)	26 (21)	3.9
	Pulmonary disease	23 (7.1)	13 (7.3)	0.8	10 (8.1)	9 (7.3)	3.1
	Biliary tract infection	83 (25.8)	28 (15.8)	22.8	26 (21)	21 (16.9)	9.2
	Acute pancreatitis	55 (17.1)	25 (14.1)	7.9	19 (15.3)	16 (12.9)	6.4
Number of stones	Single	163 (50.6)	76 (42.9)	15.4	81 (56.3)	75 (52.1)	1.6
	Multiple	130 (40.4)	65 (36.7)	7.4	56 (38.9)	64 (44.4)	4.8
	Uncertain	29 (9)	36 (20.3)	39.6	7 (4.9)	5 (3.5)	10
Preoperative TBIL, μmol/l		29.83 (18.2–67.93)	24.3 (13.4–54.95)	24.2	24.5 (15.88–49.6)	27.6 (15.05–59.53)	1.9
Preoperative DBIL, μmol/l		11 (4.78–38.25)	7.2 (2.6–25.2)	24.2	8 (3.53–19.58)	8.85 (3.03–32.48)	2.7
Preoperative WBC count, 109/l, mean (SD)		7.95 (3.62)	7.28 (3.03)	18.5	7.55 (2.76)	7.41 (2.81)	3.7
CBD diameter, mm, mean (SD)		15.22 (5.63)	13.9 (4.68)	23.5	14.23 (4.84)	14.31 (4.62)	1.4

Data are presented as a number (percentage) of patients or median (interquartile range) unless indicated otherwise.

Abbreviations: CBD, common bile duct; DBIL, direct bilirubin; SMD, standardized mean difference; TBIL, total bilirubin; WBC, white blood cell

### Comparison of outcomes 

The rates of postoperative complications in the 2 matched cohorts are compared in TABLE 2  . There were no notable differences between the groups in terms of total post-operative complications (P = 0.08), acute pancreatitis (P >0.99), recurrent CBD stones (P = 0.25), bile leakage (P = 0.12), CBD stricture (P >0.99), pulmonary infection (P >0.99), peritoneal effusion (P = 0.12), pleural effusion (P >0.99), gastroparesis (P >0.99), and DVT of the lower extremities (P >0.99). However, the incidence of postoperative hyperamylasemia was higher in the drainage group than in the nondrainage group (P = 0.006).

As illustrated in TABLE 3 , we evaluated the effect indicators in the 2 matched cohorts. Overall, the nondrainage group demonstrated better outcomes than the drainage group. Specifically, the nondrainage group was characterized by lower total hospitalization cost (P = 0.01), shorter operative time (P <0.001) as well as shorter total and postoperative hospital stay (both P <0.001). Additionally, the duration of postoperative antibiotic use (P <0.001) was shorter and the need for antispasmodics (P = 0.001) and analgesics (P <0.001) was less prevalent in the nondrainage group, as compared with the drainage group.

## DISCUSSION 

To our best knowledge, this is the first study involving patients in whom LCBDE was performed without any biliary or abdominal drainage. Prior to this study, we collected data from 123 patients with CBD stones who underwent LCBDE at our hospital between January 2014 and November 2020 (unpublished data). These patients were subjected to simple primary closure without any drainage. The surgery resulted in removal of all stones in the whole study group, with a mean (SD) operative time of 95.8

(25) minutes and a mean (SD) postoperative hospital stay of 2.3 (1.1) days. Two patients (1.6%) experienced postoperative complications, including 1 case of biliary infection and 1 case of biliary bleeding, both of which resolved following conservative treatment. There were no cases of bile leakage, CBD stricture, or reoperation. The preliminary data indicated that LCBDE without any drainage was associated with shorter recovery time and a low and acceptable rate of post‑operative complications. Furthermore, the complications observed in these 2 cases could not have been mitigated by the use of abdominal drainage tubes. Our preliminary study suggests that nondrainage LCBDE is safe. However, there were several limitations to our earlier research, such as significant selection bias, as we tended to choose simpler cases for nondrainage surgeries, which led to overly positive results. Additionally, the sample was too small, and even with a control group, we could not balance the confounding factors between the groups. Therefore, we conducted the present study to further investigate the safety and feasibility of nondrainage LCBDE. 

The benefit of bile duct drainage during LCBDE is debatable. In the era of laparoscopic surgery, single‑stage LCBDE is proven superior to ERCP + LC^23^ in terms of hospital stay duration, need for additional procedures, and cost‑effectiveness.^2^ Additionally, LCBDE is associated with a higher rate of complete stone clearance and a lower incidence of postoperative complications, such as pancreatitis.^24^ Therefore, LCBDE should be considered the optimal treatment choice for cholecysto‑choledocholithiasis.^24^^;^^25^^;^^26^^;^^27^ In the past, intraoperative cholangiography was used rather than cholangioscopy, but this approach did not allow for ascertaining whether the stones had been removed completely; therefore, postoperative T‑tube placement was particularly important. The T‑tube is used to alleviate biliary pressure and reduce the risk of bile leakage. More‑over, it provides support to the CBD, preventing its stricture. Additionally, the T‑tube can be used for postoperative cholangiography to detect any residual stones and provide a pathway for their removal through the tube.^28^ However, the drawbacks of T‑tube placement have become increasingly apparent. These include hindering patient postoperative mobility and gastrointestinal function recovery, causing electrolyte imbalances due to bile loss, and bringing about the potential for severe complications, such as biliary peritonitis if the T‑tube dislodges.^10^ With the wide use of intraoperative cholangioscopy and improvements in operative techniques, T‑tube still plays a significant role in specific cases, especially when CBD stricture is identified intraoperatively or when bile duct stones cannot be completely removed. However, in many uncomplicated cases, the use of a T‑tube has proven inconvenient and unnecessary.^10^^;^^13^^;^^29^ LCBDE with simple primary closure of the CBD was proven to be safe^9^^;^^30^^;^^31^^;^^32^ and might be performed as a first‑choice treatment in patients with choledocholithiasis.^8^^;^^33^^;^^34^^;^^35^

**TABLE 2:** Comparison of postoperative complications between the groups after propensity score matching

Parameter	Drainage group	Nondrainage group	*P *value
	(n = 124)	(n = 124)	
Total postoperative complications	24 (19.4)	14 (11.3)	0.08
Hyperamylasemia	19 (15.3)	6 (4.8)	0.006
Acute pancreatitis	0	1 (0.8)	>0.99
Common bile duct stone recurrence	0	3 (2.4)	0.25
Bile leakage	4 (3.2)	0	0.12
Common bile duct stricture	0	1 (0.8)	>0.99
Pulmonary infection	2 (1.6)	1 (0.8)	>0.99
Peritoneal effusion	1 (0.8)	0	0.12
Pleural effusion	0	1 (0.8)	>0.99
Gastroparesis	0	1 (0.8)	>0.99
Deep vein thrombosis	1 (0.8)	0	>0.99

Data are presented as a number (percentage) of patients.

Recently, other types of bile duct drainage, including bile duct stent insertion,^10^^;^^11^ spontaneously removable biliary stent drainage,^36^ D‑J tube drainage,^37^ pigtail J‑tube drainage,^38^ and intraoperative ENBD,^12^^;^^39^ have shown better results than T‑tube drainage and / or primary duct closure in terms of postoperative hospital stay and hospitalization cost. These methods also help alleviate biliary pressure and reduce the risk of bile leakage to some extent. However, some researchers found that antegrade stents or T‑tube insertion do not provide any added value for choledochotomy closure, and that they are associated with specific morbidity.^13^ Additionally, a study by Yin et al^28^ indicated no significant benefit of various internal or external drainage techniques for primary duct closure. Simultaneously, Lai et al^14^ demonstrated that primary closure of the CBD in LCBDE without any biliary drainage (including T‑tube, nasobiliary duct, or biliary stent) is feasible and safe in selected patients meeting specific criteria. Therefore, additional bile duct drainage may be unnecessary for most patients with CBD stones.

In patients at a high risk of bile leakage, such as those with unsatisfactory CBD sutures, severe local adhesions, or severe infection at the surgical site, abdominal drainage is conventionally used to mitigate the consequences of such leakage. However, similarly to various forms of internal and external biliary drainage (eg, a T‑tube), abdominal drainage may be unnecessary, especially if the surgery is performed by a team with vast experience in primary closure in LCBDE, and in most uncomplicated cases. The absence of abdominal drainage reduces patient discomfort, which accelerates postoperative recovery. In our study, after PSM, the use of postoperative antispasmodics and analgesics was significantly less prevalent in the nondrainage group, as compared with the drainage group. This might be due to the pain caused by the traction of the drainage

tube or gastrointestinal spasms induced by its presence in the abdominal cavity. Additionally, the operative time, total length of hospital stay, postoperative hospital stay, and duration of post‑operative antibiotic use were significantly shorter, and the total hospitalization cost was lower in the nondrainage than in the drainage group. In the nondrainage group, the duration of preventive antibiotic use generally did not exceed 48 hours postoperatively, while in the patients who underwent abdominal drainage, it was extended due to the potential risk of retrograde infection through the drainage tube, inevitably leading to some bias. Patients in the nondrainage group could generally be discharged 1 to 2 days after the surgery if blood test results were normal, whereas the individuals who underwent drainage had to wait for the drainage tube to be removed before discharge could be considered. Consequently, the hospitalization time in the drainage group was extended, which increased the hospitalization cost. The median (IQR) length of postoperative hospital stay in the nondrainage group was 2 (1–2) days, which is shorter than that reported in most previous studies^9^^;^^11^^;^^14^^;^^23^^;^^30^^;^^31^^;^^32^^;^^40^

Regarding postoperative complications, after PSM, 14 patients (11.3%) in the nondrainage group experienced complications, including 6 cases (4.8%) of hyperamylasemia, 3 cases (2.4%) of recurrent CBD stones, and 1 case (0.8%) each of acute pancreatitis, CBD stricture, pulmonary infection, pleural effusion, and gastroparesis. In the drainage group, 24 patients (19.4%) experienced complications, including 19 cases (15.3%) of hyperamylasemia, 4 (3.2%) cases of bile leakage, 2 cases (1.6%) of pulmonary infection, and 1 case (0.8%) each of peritoneal effusion and DVT. Most complications (97.18%) were classified as Clavien–Dindo grade I or II. The patients with postoperative hyperamylasemia did not require clinical intervention. The individuals with acute pancreatitis, mild CBD stricture, pulmonary infection, and gastroparesis recovered after conservative treatment. Among the 4 patients with bile leakage, 3 were classified as grade A and did not require clinical intervention, while 1 was classified as grade B and recovered smoothly after ultrasound‑guided percutaneous drainage and anti‑infective treatment. One patient with peritoneal effusion and 1 individual with pleural effusion required additional drainage. Only 3 patients with recurrent CBD stones and 1 patient with DVT required additional surgical treatment. In both groups the complication rates were higher than reported in most previous studies. This is because many studies^6^^;^^9^^;^^11^^;^^14^^;^^30^^;^^31^^;^^39^ either do not focus on postoperative hyperamylasemia or do not consider it a postoperative complication.^39^ According to the Clavien–Dindo definition, complications should be understood as any deviations from the normal postoperative course, including asymptomatic complications. Hence, we classified postoperative hyperamylasemia as a complication. Our results indicate that the total and individual complication rates (except for hyperamylasemia) did not differ significantly between the nondrainage and drainage groups, suggesting that LCBDE without drainage does not increase the risk of postoperative complications and is equally safe and effective in eligible cases. The incidence of postoperative hyperamylasemia in the drainage group was significantly higher than in the nondrainage group (15.3% vs 4.8%). This is likely due to the fact that the abdominal drainage tube is a foreign body causing local inflammation and irritation in the abdominal cavity. The tube might also stimulate the gastrointestinal tract, leading to spasms and promoting the release of pancreatic enzymes, which is why longer operative time in the drainage group may lead to prolonged pneumoperitoneum and anesthesia time during surgery. However, these are only suppositions, and further investigation is needed to explore the potential risk factors for postoperative hyperamylasemia following LCBDE.

**TABLE 3:** Comparison of effect indicators between the groups after propensity score matching

Parameter	Drainage group (n = 124)	Nondrainage group (n = 124)	t/χ2	*P *value
Total hospitalization cost, CNY	25 085.55 (22 046.83–29 372.33)	23 546.29 (21 735–26 163.33)	2.499	0.01
Total length of hospital stay, d	8 (6–11)	6 (4–8)	4.417	<0.001
Length of postoperative hospital stay, d	3 (2–5)	2 (1–2)	8.567	<0.001
Operative time, min	120 (95–145)	110 (90–125)	2.791	0.005
Duration of postoperative antibiotic use, d	3.5 (3–5)	2 (1–3)	7.366	<0.001
Postoperative doses of analgesics, n	2 (1–4)	1 (0.25–2)	4.296	<0.001
Postoperative doses of antispasmodics, n	2 (0–5.75)	1 (0–2)	3.175	0.001

Data are presented as median (interquartile range). Abbreviations: CNY, Chinese yuan

To ensure clinical safety, we recommend that nondrainage LCBDE be performed by surgical teams experienced in LCBDE and primary closure of CBD. Only when CBD stones are completely extracted, the lower end of the CBD is free of stenosis, and bile excretion is clear can primary closure of CBD be performed during LCBDE. It is essential to minimize biliary leaks to ultralow levels so as to avoid abdominal drainage. In our study, when opening the CBD, an electrocautery hook was typically used to make a longitudinal incision of 1 to 1.5 cm in the anterior wall. The incision length should be sufficient to allow for choledochoscope entrance and subsequent stone removal, but it should not be excessively large. The choledochotomy incisions were primarily closed with interrupted sutures using Vicryl 3–0 or 4–0 suture material. After stone removal, sutures were first placed at the upper and lower ends of the incision (slightly beyond the incision site). The stitches should be spaced 1 to 1.5 mm apart, with a margin of 1 to 2 mm from the incision edge, ensuring full‑thickness, interrupted mucosa‑to‑mucosa sutures. If the serosa of the hepatoduodenal ligament was prominent, it had to be sutured and reinforced. During surgery, gauze and suction were repeatedly used to confirm the absence of significant bile leakage from the suture line. Special attention should be paid to maintaining appropriate spacing between stitches, ensuring adequate knot tension, and avoiding bile leakage from needle holes caused by repeated needle insertion.

Our study has certain limitations. Due to its nonrandomized, retrospective design, inherent confounding factors exist. Although we used PSM to effectively reduce the confounding bias, some unaccounted‑for confounders may still be present. Additionally, certain outcome measures, such as the length of postoperative hospital stay and the duration of postoperative antibiotic use, may be discrepant due to variations in clinical practices and subjective judgments among different surgeons. Moreover, we did not use conventional pain scores41 to evaluate postoperative pain levels. Instead, we used the more objective measures of postoperative usage of antispasmodics and analgesics to reflect patient pain levels. However, differences in empirical medication practices among different surgeons may give way to additional bias. In our study, the procedures were performed by surgeons with varying levels of expertise, from attending to chief physicians. This shows that performing LCBDE without any drainage primarily requires an understanding of the importance of accelerated recovery postsurgery, and willingness to challenge the traditional approach involving routine drainage. From January 2014 to November 2020, we completed 123 nondrainage LCBDE surgeries, while from December 2020 to December 2023, 177 such procedures were performed. The increase in the volume of this surgery reflects a shift in surgical philosophy from a traditional standpoint to an approach focused on accelerated recovery. Future research in this field requires larger sample sizes, multicenter participation, and prospective study designs to draw more reliable conclusions.

## CONCLUSIONS 

LCBDE without any kind of biliary or abdominal drainage is a safe and feasible method for eligible patients, provided it is performed by experienced surgical teams.
